# Digital bonds: patient and therapist factors influence telehealth rapport building in speech-language services

**DOI:** 10.3389/fpsyg.2025.1612803

**Published:** 2025-11-27

**Authors:** Ying Hao, Hyejin Park, Jaret Webb

**Affiliations:** 1 School of Chinese Language and Literature, Nanjing Normal University, Nanjing, China; 2 Department of Communication Sciences and Disorders, University of Mississippi, Oxford, MS, United States; 3 Baptist Health Medical Center, Little Rock, AR, United States

**Keywords:** telehealth, rapport building, speech-language pathologist, telehealth experience, digital literacy

## Abstract

**Introduction:**

Rapport building is challenging in pediatric telehealth speech-language services, yet factors influencing it remain unclear. The study specified how patient and clinician factors contributed to the issue.

**Methods:**

Generally, the two disorders reflect different levels of behavioral, sensory, and cognitive challenges, and the two ages index different developmental stages. This study surveyed 207 speech-language pathologists (SLPs) about three aspects (i.e., importance, strategy use, and achievement) of rapport when working remotely with children diagnosed with speech sound disorders (SSD) or autism spectrum disorder (ASD) at two ages (0–3 years, 4–8 years). Clinician factors included clinician age, telehealth experience, and digital literacy.

**Results:**

Regarding patient factors, SLPs rated the ASD group higher importance, more strategy use, but lower achievement than the SSD group. Clinicians reported more strategy use and lower achievement of rapport when serving younger children, but a main effect of patient age was not found in the perceived importance of rapport. Regarding clinician factors, older SLPs tended to place higher importance, use more strategies, and feel more achieved on telehealth rapport than younger SLPs. While SLPs without telehealth experience reported similar levels of strategy use for SSD and ASD groups, those with experience, regardless of the diversity of disorder-age combinations, used strategies more frequently for the ASD group. Finally, digital literacy was significantly related to SLPs’ perceived levels of achievement.

**Conclusion:**

Overall, these findings underscore the importance of both patient and clinician factors when establishing rapport in telehealth, which may be implicational for other professionals who provide services to individuals with disabilities.

## Introduction

Telehealth employs a range of telecommunication technologies, such as real-time audiovisual conferencing and asynchronous electronic transmissions of therapy materials, which allows therapists to reach their patients from distance (World Health Organization, 2017). Different terms are used when referring to remote communication and its uses to serve patients, for example, telehealth, telemedicine, and telepractice. Commonly, telehealth is a broader term which can be used to describe the synchronous or asynchronous variations of videoconferencing and electronic transmission of therapy services. In the opposition, telemedicine is more focused on the delivery of medical services, which is in the process of being phased out in favor of telehealth, as the term likely restricts the scope to services involving only medical management (Center for Connected Health Policy, n.d.). The American Speech-Language Hearing Association (ASHA) adopted the term telepractice to emphasize that speech-language/audiology services delivered remotely span a range of educational settings, in addition to healthcare settings (American Speech-Language-Hearing Association, n.d.-a). Currently, as telehealth is being used most widely by various healthcare disciplines (e.g., dentistry, counseling, physical and occupational therapy, home health, chronic disease monitoring and management, and disaster management), it is used throughout the current study.

Telehealth brings about benefits by offering increased access to services for rural and under-served patients, and increased flexibility of time for both patients and therapists. It is easier to involve caregivers, interpreters, or remote specialists in real time. It also facilitates clinician training and supervision, supports asynchronous tools (e.g., recorded practice, messaging) to extend therapy between sessions, and enhances service resilience during public health emergencies (Zilliacus et al., 2010). Despite its many advantages, the service delivery method contains unique challenges which may not be as evident as in conventional in-person therapy (Tucker, 2012a). A commonly cited challenge of telehealth is building rapport between therapists and patients, possibly due to reduced nonverbal channels, limited visual field, and technical problems. Rapport has been defined as the establishment and maintenance of an interactive, harmonious, and communicative relationship between the therapist and the patient (Pattison and Powell, 1989). Recent studies share a similar definition, that is, both sides share mutual feelings of trust, respect, connection, and agreement on targeted goals (Freckmann et al., 2017; Duffy et al., 2023).

### Perceptions of rapport building in telehealth speech-language services

Speech-language pathologists (hereafter SLPs) have been increasingly utilizing telehealth during the COVID-19 pandemic (Hao et al., 2021; Sylvan et al., 2020) and continued to do so in the post-pandemic era (Van Echo et al., 2023). While delivering telehealth services, rapport has been widely perceived by SLPs to be challenging (e.g., Akamoglu et al., 2018; Retamal-Walter et al., 2022a). This difficulty can be especially pronounced in pediatric settings, where children’s limited cooperation could impede engagement and participation.

Three clinician-reported aspects were identified from the extant literature, aligning with the notion that rapport is multi-dimensional rather than unitary (Tickle-Degnen and Rosenthal, 1990). The first aspect relates to the perceived importance of rapport building. Tucker (2012b) and Akamoglu et al. (2018) conducted interviews with SLPs who had varying levels of telehealth experience, primarily in school settings. A majority of the participants emphasized the importance of rapport building in the success of telehealth services, citing challenges such as the lack of physical contact or proximity with students and the increased effort and collaboration required. Clinicians also reported that they prioritize a harmonious relationship with the family and the child, so clients are more likely to buy-in and carry over outside the therapy sessions. In addition, it was reported that training on rapport building skills is necessary prior to the start of telehealth, especially for children with more sensory and behavior issues.

The second aspect emerging from the literature, achievement of rapport building, was rated lower in telehealth than in-person therapy. Tucker (2012a) conducted a survey among 170 school-based SLPs in one northeastern state of the United States. Most participants disagreed with the statement that *rapport between the SLP and student can be established during speech-language telehealth just as strongly as during in-person speech-language therapy*. It is important to note, however, that only seven of these SLPs (4.1%) had experience with telehealth at the time of the survey, while the others had only conducted in-person therapy. Hines et al. (2015) noted that SLPs had reservations before the start of telehealth services but developed more positive views after gaining experience with it. Additionally, the speed at which SLPs build rapport in telehealth, which belongs to the achievement of rapport, was reported to take longer in telehealth than expected when compared to face-to-face sessions (Pitt et al., 2018; Anderson et al., 2015).

The third aspect, strategies used by SLPs to facilitate remote rapport building, has been understudied. By referring to findings from other disciplines (e.g., psychology, nursing), some common strategies were summarized. Verbal cues, such as reflections of emotions, restatements, reinforcements, descriptions, and explanations, were believed to form a stronger emotional bond, hold interests and focus, and allow the therapist more control during telecommunication (Sucala et al., 2013). Nonverbal cues are important during in-person interactions, for instance, smiling, directed gazing, head nodding, leaning forward (Tickle-Degnen and Rosenthal, 1990). In telehealth, adaptations of nonverbal cues were reported to be used by SLPs (Grillo, 2017), but specifics in regards to these adaptations were unclear to the reader. One possibility is that SLPs could use reduced nonverbal cues because of a restricted view for patients to observe these cues. The other possibility could be that SLPs exaggerate their use of nonverbal cues to compensate for the limited view, making nonverbal cues more obvious for patients to detect (Retamal-Walter et al., 2022b). Beyond verbal and nonverbal cues, e-helpers (e.g., teachers or parents sitting with the student to facilitate the online session) have been brought up widely in the literature. An e-helper could aid the student by logging on at scheduled time, obtaining and maintaining attention, and working with the student to complete the homework assigned (Tucker, 2012b).

In light of the view that rapport is dimensional, the three aspects map onto Tickle-Degnen and Rosenthal (1990)’s framework: the importance and achievement aspects reflect the affective facet of the bond (i.e., the feelings of the participants during the experience of rapport), while strategy use captures its behavioral facet (i.e., actions that express the affective connection between the participants). The two facets are interdependent rather than strictly separable. Higher perceived importance likely motivates clinicians to deploy more rapport-building strategies, but greater strategy use does not guarantee a high level of rapport achievement (Wolk et al., 2016; Zilcha-Mano et al., 2016). In some cases (e.g., children who have severe behavioral or sensory difficulties), clinicians may use many strategies yet still report low levels of achievement. This potential mismatch underscores the need to consider client and clinician factors when evaluate rapport, a point that will be expanded in the next section.

### Potential influences of patient and clinician factors

Bordin’s (1979) working alliance framework is a classic and highly influential model that defines psychotherapy alliance in terms of three components, including bond, tasks, and goals. While both patients and clinicians need to agree on the targeted goals and tasks, the bond of the two parties fosters open communication and emotional engagement. Rapport building, the focus of the current study, occupies the bond component, the interpersonal connection, warmth, trust, and engagement that makes collaborative work possible. Bond (or rapport) emerges from a good “fit” between patient and therapist, necessitating the readiness from both parties. In pediatric speech-language services, patient-side readiness to form a bond is largely shaped by a child’s developmental level and severity of challenging behaviors, while therapist-side attributes are related to clinical experience and their age, which may determine how well the clinician can echo and scaffold the child’s needs in treatment. Crucially, the digital environment adds layers by incorporating interactivity and adaptability of online communication. In particular, clinicians’ telehealth experience and digital literacy influence the formation of a strong bond remotely (Tremain et al., 2020). Overall, the bidirectional fit, patient readiness and therapist responsiveness, supports agreement on tasks and goals and thus strengthens the overall working alliance, which contribute to treatment outcomes. However, existing research provides only a broad understanding of this issue, which did not address contextual factors relevant to patients and therapists that may significantly affect telehealth rapport in pediatric speech-language services.

#### Patient factors: child diagnosis and age

As reported by therapists in interviews, children who are not likely to engage during telehealth have severe behavioral, sensory, and cognitive issues (e.g., Akamoglu et al., 2018; Retamal-Walter et al., 2022b). These symptoms are in general related to a child’s diagnosis and age. For example, children with autism spectrum disorder (ASD) are likely harder to be engaged compared to children with speech sound disorder (SSD). Both disorders make up the bulk of an SLP’s caseload (Broomfield and Dodd, 2004; Gillon et al., 2017): the overall prevalence of ASD is estimated to be about 3.2% (1 out of 31 children) (Shaw et al., 2025), and the prevalence of SSD is found to be even higher, around 3.5% (Eadie et al., 2015; Hambly et al., 2013).

To be more specific, the two disorders make a difference in how easily a digital bond forms. ASD affects the way a child communicates due to its common symptoms. Rigid and repetitive language, narrow interests and exceptional abilities, uneven language development, and poor nonverbal conversation skills, are all contributing factors that may affect how easy it is for one to build rapport with clients who have ASD (American Psychiatric Association, 2013; National Institute of Deafness and Other Communication Disorders, 2020). Effective rapport building requires clinicians to address these ASD-specific behaviors. In contrast, the behaviors of those with SSD are not as limiting. Most of the behaviors displayed by children with SSD are typically-developing behaviors and are on track with those of children who do not have SSD.

While the ASD group generally represents children with more severe behavioral, sensory, and cognitive issues, the SSD group represents those with mild or no such behaviors. As it was not feasible to exhaustively assess all disorders that SLPs may encounter in clinical settings, the two disorders were chosen based on their distinct behaviors, which may influence how rapport is established in telehealth. The contrast in their behavioral presentations served as a basis for examining how SLPs might adapt their perceptions of rapport across different types of clients. It is important to note that there was no intention to ignore the heterogeneity within each disorder. Rather, the focus was on identifying general patterns, not individual differences.

Differing levels of assistance required by ASD and SSD may alter dynamics among key stakeholders, including children, parents, clinicians, etc., thereby affecting rapport building in remote sessions. For example, children with SSD can be more independent and have more opportunities to directly interact with the clinician to build a strong rapport. However, children with ASD may heavily rely on their parent(s)/e-helper to communicate with the clinician and to assist in completing the tasks in a telehealth session. This reduces opportunities for direct clinician-child interaction and increases the burden on parents, which may undermine the formation of a strong therapeutic bond between the clinician and the child.

In addition, the diagnosis may affect patients’ digital literacy differently, which in turn influences the formation of a digital rapport. Digital literacy is defined as the ability to adapt, access, and learn technology to contribute to own community (International Society for Technology in Education, 2007; Wing, 2006). Due to the impaired communication and cognitive skills, children with ASD in general are expected to have lower digital literacy than children with SSD. Though evidence indicates telehealth can be effective for children with ASD (Christopoulou et al., 2022; Sundarrajan and Franco, 2024), it was recommended that support personnel be included for troubleshooting and that backup modalities be secured to ensure reliable therapy delivery for this population (Boisvert et al., 2010). Typically, parents take the role of the support personnel or e-helper, whose digital literacy skills have an influence on rapport in telehealth.

Child age broadly indexes the developmental level, which may influence a child’s capacity to form a digital bond. The first 3 years is the most intensive period for acquiring speech and language skills (Hoff, 2013; National Institute of Deafness and Other Communication Disorders, 2017), when early intervention services occur in the home setting (American Speech-Language-Hearing Association, n.d.-b). In contrast, at later developmental stages (e.g., ages 4–8 years), children experience their first introduction to educational settings. While at the younger age communication mainly takes place between the child and the parent, at the older age it shifts to communication among multiple communication partners such as teachers and classmates. Children who are older develop longer attention spans, resulting in increased engagement and inquisitive learning. In contrast, children who are younger possibly pose a greater threat to remote rapport due to short attention spans, requiring the SLP to implement more strategies in order to engage the client and to keep them focused. In addition, younger children tend to shy away from strangers and unfamiliar settings, whereas older children’s language skills and attention spans are more mature (Woods et al., 2013), resulting in increased engagement in different settings.

Parent–child bonding patterns vary by diagnosis and developmental level, which may shape how successfully a clinician establishes rapport remotely with the family. The first 8 years of life are the most crucial time for the parent–child relationship, influencing the quality of the family environment and the child’s development (Breiner et al., 2016). However, family members with severe disabilities, such as ASD, may negatively impact the family relationship and introduce an additional source of stress (Schieve et al., 2007). Because rapport is typically built on a positive environment among team members (e.g., SLP, child, and parent or support personnel), the additional time and assistance that parents need to provide in telehealth likely increases parental stress and burden. This may impede clinicians from building a successful rapport with the family, particularly for parents who have lower digital literacy.

#### Clinician factors: clinician age, telehealth experience, and digital literacy

Based on the existing literature, clinician factors mainly included their age, telehealth experience, and digital literacy. Tucker (2012a) looked at how the overall work duration affected perceptions of telehealth use. A group of SLPs (*n* = 25) with 1–5 years of work experience expressed greater interest in the use of telehealth, while a group of SLPs (*n* = 74) with 25 + years of experience showed reduced interest. The finding was interpreted in relation to clinician age. SLPs who were older displayed lower acceptance of telehealth than SLPs who were younger. Although the divided perceptions were not about rapport building per se, they could influence how SLPs perceive rapport building. In addition, telehealth experience appears to significantly shape SLPs’ perceptions of telehealth. Those who have adopted telehealth often develop more positive views, whereas SLPs with no telehealth experience may maintain more negative perceptions (Hines et al., 2015).

Digital literacy refers to the competence in using digital technologies, such as tablets, smartphones, apps, the internet, and digital cameras, independent of any specific health-related purpose (Longhini et al., 2022).^1^ Computer glitches and/or internet disconnection could cause sound distortion and overlapped segments of speech, making patients frustrated within their sessions (Retamal-Walter et al., 2022b). This highlights the importance of clinicians’ digital literacy in building rapport with their clients in telehealth settings. Limited digital literacy among therapists could hinder child and family participation and could make rapport building difficult. If this occurs, individual therapist’s levels of rapport achievement could be lower.

### The current study

The study explored the influences of patient and therapist factors on SLPs’ perceptions of rapport with pediatric patients in telehealth. Regarding patient factors, it surveyed digital bond when serving children with different diagnosis (i.e., ASD or SSD) and different ages (i.e., 0–3 years old or 4–8 years old). Regarding therapist factors, it assessed how SLPs’ digital literacy, age, and telehealth experience correlated with perceptions of rapport in telehealth. Rapport was treated as a multi-dimensional construct and was evaluated from three aspects (i.e., importance, levels of achievement, and strategy use).

Drawing on Bordin (1979)‘s framework and the extant literature in telehealth speech-language services, we derived hypotheses addressing patient and clinician factors, respectively. Generally, patients who are younger or who present greater behavioral and sensory challenges are expected to cause more difficulties for clinicians to build rapport remotely. Thus, clinicians were predicted to perceive digital rapport as more important, apply more strategies, but feel less achieved when working remotely with younger children and the ASD group, compared to older ones and the SSD group. Regarding clinician factors, those who are younger in age, more experienced with telehealth, and more skilled in digital literacy would perceive rapport more important, use more strategies, and feel more achieved when building rapport remotely. Regarding correlations among the three aspects of rapport building, we predicted that clinicians’ ratings of perceived importance would be positively and significantly associated with reported strategy use, whereas strategy use would not be significantly correlated with their achievement of rapport.

## Methods

### Participants

Ethical approval was obtained from one of the affiliated universities. Eligible SLPs were those with experience treating children with both disorders, ASD and SSD, and at least one age range, 0–3 or 4–8 years old. As the two age ranges typically relate to different work settings (e.g., Early Intervention versus School), it is hard to have participants to cover both ages. Participants were not required to have experience in telehealth in order to participate in the study, allowing us to investigate how varying levels of telehealth experience affected participants’ perceptions of rapport building. Finally, participants were informed that ASD or SSD had to be the primary diagnosis, rather than secondary to any other condition.

A power analysis was conducted to determine the minimum number of participants that should be recruited. Generalized Linear Mixed Modeling (GLMM) was planned to be used for data analysis (specified below). We implemented power analysis for repeated measures ANOVA which is regarded as most similar to the GLMM.^2^ With a significance criterion of *α* = 0.05, power = 0.95, and the effect size of 0.25, a minimum of 126 SLPs were required. Response rate was unknown to us, so we were being conservative (i.e., 1%). Therefore, invitations were sent to 350 SLPs in each of the 50 states (17,500 invitations in total), which could bring about 175 participants (above the minimum sample size requirement). In practice, there were nine states that did not have 350 SLPs (i.e., Delaware, Hawaii, Alaska, Montana, Wyoming, North Dakota, South Dakota, Rhode Island, Vermont). In this case, survey invitations were sent to all eligible SLPs matching the search criteria for that state. In total, 16,165 invitations were sent out, with a total of 221 SLPs participating in the survey, yielding a response rate of 1.4%.

Dissemination of survey started from the selection of participants using the ASHA Community Directory, which included a private message feature for communication among ASHA members. Filters were applied to help target eligible SLPs, including *autism spectrum disorder, articulation disorders*, and *phonological disorders*. An initial screening link was sent to each potential eligible SLP, which asked if the participant was a licensed SLP who had experience working with children with ASD and SSD. In addition, SLPs were informed via the screening link that incentives ($10 Amazon e-gift card) would be provided to randomly selected participants who completed the survey. If an SLP believed that they were eligible, they were asked to leave their names and email addresses, which were used to generate a unique personal link to take the survey. This approach minimized invalid responses and prevented any ineligible participants from taking the survey in order to gain incentives. Both the initial screening survey and the subsequent research survey were developed and delivered through Qualtrics.

Consent was obtained from the participants before they proceeded to the research survey. Survey responses were collected from October to December 2021. Once the unique survey link was sent, the participant was given 2 weeks to complete it. A reminder email was sent approximately 1 week after the link was initially sent to remind interested SLPs to complete the survey. At the end of December 2021, one final reminder was sent to all interested SLPs who had not initiated or completed the research survey. In January 2022, the survey was closed, and incentives were sent.

### Research survey

The study employed an e-survey, following Consensus-Based Checklist for Reporting of Survey Studies (CROSS) (Sharma et al., 2021). The research survey was organized into four sections. The first section included demographic questions (i.e., gender, age, ethnicity, etc.), years holding CCC-SLP certification, years of using telehealth in a pediatric setting, and prior telehealth training. In addition, SLPs were asked to report their telehealth caseloads and their entire working history caseloads, with regard to each of the disorders (ASD and SSD) and each of the age ranges (0–3 and 4–8). Altogether, the information in this section helped define the sample of SLPs. Digital literacy was put into two questions (i.e., internet competence and computer competence), as they are different concepts. Internet competence refers to their ability to connect to the internet and troubleshoot common connectivity issues. Computer competence refers to their ability to work, maneuver, or troubleshoot issues related to the use of a computer, tablet, smart phone, and other devices.

In the remaining three sections, the three aspects of rapport building in telehealth, including perceived importance, strategies employed, and perceived achievement, were evaluated. The questions within each section were based on the literature that have been reviewed. Each question asked the participant to notate their response for one disorder (ASD or SSD) and for one age range (0–3 or 4–8). Rankings were placed on a five-point scale, with lower values representing lower perceptions and higher values representing higher perceptions. See Appendix 1 for the complete survey questionnaire.

After the questions were drafted, the authors collected perspectives and feedback from two researchers who had published in the area of telehealth and two seasoned SLPs who had experience with the two disorders and the two ages via telehealth. They returned the survey with comments and edits, focusing on improving the clarity of the questions and the rewording of vague language. For example, per a comment from one expert, *caseload* was specified as *caseload for the entire working history* versus *caseload for telehealth only*. The survey questions were finalized when the comments and edits were fully addressed. Upon the completion of this step, two graduate students majoring in speech-language pathology tested the initial screening survey and the follow-up research survey, ensuring that both surveys and their links could be used reliably. The process provides content and face validity and pilots the survey before it was disseminated to a large cohort.

## Results

### The current sample of SLPs

Eligibility criteria were applied to finalize the sample: eight participants were excluded as their responses to the survey were incomplete; six participants were excluded as they had experience with neither disorder throughout their entire career. Altogether, 14 participants were excluded, yielding 207 eligible participants with complete data being included in the analysis.

Table 1 presents the results of the background section of the survey. In the current sample, most SLPs were female and Non-Hispanic White, with around 80% between 30 and 59 years old. Most worked in schools, followed by private practices, early intervention, and non-residential health care, while each of the remaining settings accounted for fewer than 5%.

**Table 1:** Participant background information (*n* = 207).

Characteristics	Number	Percentage (%)
Gender		
Female	*201*	97.1
Male	*5*	2.4
Other (no-binary)	*1*	0.5
Age		
20–29	*16*	7.7
30–39	*57*	27.5
40–49	*58*	28.0
50–59	*45*	21.7
60–69	*26*	12.6
70–79	*5*	2.4
Ethnicity		
Asian	*5*	2.4
Black or African American	*8*	3.9
Hispanic	*2*	1.0
Non-Hispanic White	*184*	88.9
Mixed or Other (e.g., Non-Hispanic White and Native American, Jewish, European)	*8*	3.9
Highest education		
Master’s degree	*200*	96.6
Doctorate degree (PhD, EdD, or SLPD)	*7*	3.4
Work setting (participants could choose more than one)		
School	*109*	52.7
Early intervention	*49*	23.7
College/University	*9*	4.3
Hospital	*11*	5.3
Residential health care	*4*	1.9
Non-residential health care	*20*	9.7
Private practice	*68*	32.9
Other (e.g., behavior day treatment, Head Start)	*7*	3.4
Geographic region of practice^a^		
Northeast	*35*	16.9
Southeast	*47*	22.7
Southwest	*22*	10.6
Midwest	*53*	25.6
West	*50*	24.2
Rurality of practice^b^		
Rural	*42*	20.3
Urban	*140*	67.6
No data found	*25*	12.1
Years of holding certificate		
0–10 years	*62*	30.0
11–20 years	*56*	27.1
21–30 years	*52*	25.1
31–40 years	*28*	13.5
More than 40 years	*9*	4.3
Years spent using telepractice in a pediatric setting		
Less than 1 year	*52*	25.1
1–2 years	*123*	59.4
2–3 years	*15*	7.2
More than 3 years	*17*	8.2
Telepractice training		
Formal training (e.g., courses in college or CEU)	*78*	37.7
No formal training (e.g., self-taught)	*121*	58.5
No training	*8*	3.8
Internet competence (e.g., dealing with internet disconnection)		
1. Not at all competent	*1*	0.5
2. Low competence	*5*	2.4
3. Neutral	*18*	8.7
4. Competent	*131*	63.3
5. Very competent	*52*	25.1
Average (*SD*)	*4.0 (0.7)*	
Computer competence (e.g., computer, tablet, smart phone)		
1. Not at all competent	*0*	0
2. Low competence	*2*	1.0
3. Neutral	*13*	6.3
4. Competent	*122*	58.9
5. Very competent	*70*	33.8
Average (*SD*)	*4.0 (0.6)*	

a Based on the state reported by an SLP, researchers referred to National Geographic (O’Connor, 2012) to categorize the SLP into one of the five regions.

b Based on the county reported by an SLP, researchers referred to Health Resources and Services Administration (Department of Health and Human Services, n.d.) to define whether the SLP was practicing in a rural or urban area.

Participants reported their state and county of practice, allowing researchers to determine geographic region and rurality. Using National Geographic’s boundaries (O’Connor, 2012), participants were identified in all five U.S. regions (Northeast, Southeast, West, Southwest, Midwest). Rurality status was determined through Health Resources and Services Administration database (Department of Health and Human Services, n.d.), and 12.1% of counties were unverifiable. Among the verified counties, over three-fifths of SLPs practiced in urban areas, and the remainder practiced in rural areas.

Most participants had held certification for 0–10 years, 11–20 years, or 21–30 years. By contrast, only 15.4% had provided telehealth in pediatric settings for more than two years. Possibly relating to the overall short experience with telehealth, more than half (58.5%) had no formal training on telehealth. Meanwhile, SLPs rated both their internet competence and computer competence^3^ at 4 out of 5, suggesting competence in digital literacy.

**Figure 1:**
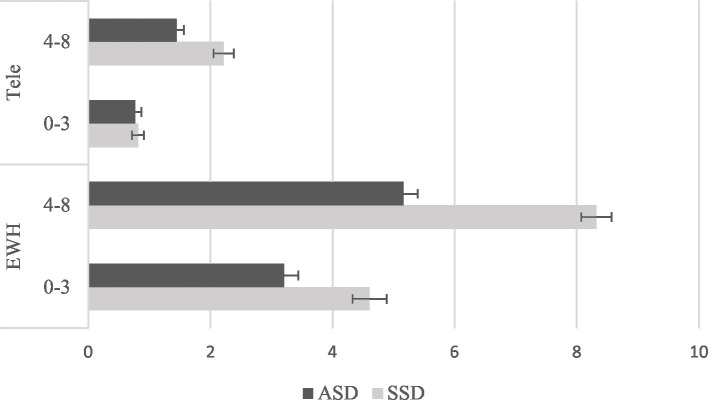
Participating SLPs’ caseloads with the two diagnoses and ages, separating entire work history and telehealth only. Tele refers to telehealth. EWH refers to entire work history. Caseload: 0 = no experience, 1 = 1–10 children, 2 = 11–20 children, 3 = 21–30 children, 4 = 31–40 children, 5 = 41–50 children, 6 = 51–60 children, 7 = 61–70 children, 8 = 71–80 children, 9 = 81–90 children, 10 = 91–100 children, 11 = 100 + children.

Figure 1 presents the reported caseload demographics for the two disorders and ages. They were separated into four categories: (1) entire work history for 0-3-year-olds, (2) entire work history for 4–8-year-olds, (3) telehealth only for 0-3-year-olds, and (4) telehealth only for 4–8-year-olds. Overall, participating SLPs reported a higher caseload of SSD than ASD and a higher caseload of 4-8-year-olds than 0-3-year-olds for both entire work history and telehealth only. Regarding telehealth, among the 207 participants, 54 (26.1%) had experience across all the four categories, 32 (15.5%) in three of the four categories, 82 (39.6) in two, 17 (8.2%) in only one, and 22 (10.6%) had no telehealth experience in any of the four categories.

### Instrument validation

The telehealth rapport building instrument initially included eight questions (three assessing perceived importance, three assessing strategy use, and two assessing perceived achievement). Each question was completed separately in four contexts (SSD at 0–3, SSD at 4–8, ASD at 0–3, ASD at 4–8), resulting in 8 × 4 = 32 items. See Appendix 1 for details. The eight questions were based upon a review of the extant literature. Expert review was conducted after the questions were drafted. Two experienced telehealth researchers and two seasoned telehealth clinicians evaluated each of these questions for relevance and clarity, and they further recommended changes in wording (also see “Research Survey” section). This process provided content and face validity.

Based on the data from the 207 participants, Cronbach’s *α* for the 32 items was 0.85 (McDonald’s *ω* = 0.86), indicating good internal consistency. Confirmatory factor analysis (CFA) was conducted on three latent factors (the three aspects of telehealth rapport building). The mean score of the four context-specific responses was used for each question to reduce strong context clustering. One question (Q17 in Appendix 1) exhibited a weak loading and thus was removed. The final model therefore included seven aggregated questions. All the indicators loaded significantly on their intended factors (moderate to strong standardized loadings ranging from 0.435 to 0.949). Fit indices were mixed but overall consistent with the three-factor interpretation: CFI = 0.927 (acceptable), TLI = 0.860 (borderline), RMSEA = 0.086 (90% CI [0.047, 0.127], moderate), *χ*^2^(11) = 27.953, *p* = 0.003 (highly sensitive to sample size), AIC = 2811.775. For comparison, a single-factor CFA on the seven aggregated questions fit substantially worse: CFI = 0.532 (poor); TLI = 0.298 (poor); RMSEA = 0.193 (90% CI [0.162, 0.225]) (poor); *χ*^2^(14) = 122.026, *p* < 0.001; AIC = 2899.849. Loadings were weak to moderate ranging from 0.078 to 0.688. Taken together, these results offer support for the three-factor structure while indicating room for improvement.

### Influences of patient and clinician factors

The analysis was conducted using R Version 4.4.3 (R Core Team, 2025) and the “ordinal” package (Christensen, 2023). We fitted cumulative link mixed models (CLMMs) with a logit link function. The model had the maximal random effects structure justified by the data that would converge, including a random intercept for participants. Items were not treated as random effects, as they represent different aspects of rapport building, rather than interchangeable stimuli. We began with the most complex by-participant random effects structure (1 + child_disorder + child_age | participant) and then sequentially reduced it to the simplest one (1 | participant). The fitted models were compared in terms of AIC, with a smaller value indicating a better model fit. This was supplemented by likelihood ratio tests conducted to determine whether the inclusion of a predictor significantly improved the model fit.

Independent variables included patient factors and clinician factors. Patient factors, child disorder and age, were treated as within-clinician repeated measures.^4^ Disorder and age were coded as categorical (SSD = 1 and ASD = 2; 0–3 years = 1 and 4–8 years = 2). Three clinician factors were included. Clinician age (20–39 = 1, 40–59 = 2, 60–79 = 3) and telehealth experience were categorized (no telehealth experience = 1; lower diversity (worked with 1 or 2 diagnosis-age groups) = 2; higher diversity (worked with 3 or 4 diagnosis-age groups^5^) = 3). All the categorical factors were dummy-coded with 1 set as the reference category. As scores of internet competence (Q11) and computer competence (Q12) were highly correlated (*r* = 0.767, *p* < 0.01), the average scores were entered into the model, indexing overall digital literacy. Interactions were included in the model, including two-way interactions of child diagnosis and child age, clinician experience diversity and child diagnosis, clinician experience diversity and child age, and a three-way interaction among clinician experience diversity, child diagnosis, and child age. Dependent variables were responses to questions regarding perceived importance, strategy use, and achievement of rapport building in telehealth, respectively. Appendix 2 displays means and standard deviations for each question related to the three aspects, distinguishing disorder-age groups.

Regarding the perceived importance of rapport building in telehealth, the analysis were based on a model including a by-participant intercept and child_disorder slope (AIC = 3729.85, *χ*^2^(2) = 9.16, *p* = 0.01). It was shown that ASD was associated with higher ratings than SSD, with an effect that approached significance (*β* = 0.43, SE = 0.23, z = 1.93, *p* = 0.054). Clinician age was positively associated with higher perceived importance of rapport: relative to clinicians aged 20–39 years, clinicians aged 40–59 years (*β* = 0.393, SE = 0.190, z = 2.064, *p* = 0.039) and 60–79 years (*β* = 1.389, SE = 0.287, z = 4.833, *p* < 0.001) showed higher odds of endorsing greater importance. Child age, clinician telehealth experience, clinician digital literacy and all tested interactions were not significant.

Regarding the achievement of rapport building in telehealth, a model with by-participant intercepts and random slopes for child_disorder and child_age provided the best fit (AIC = 3709.6, *χ*^2^(3) = 8.82, *p* = 0.03). Achievement ratings were lower for children with ASD than children with SSD (*β* = −0.74, SE = 0.22, *z* = −3.29, *p* < 0.01), higher for older children than younger children (*β* = 1.15, SE = 0.22, *z* = 5.30, *p* < 0.01), and higher among clinicians with better digital literacy (*β* = 0.60, SE = 0.21, z = 2.85, *p* < 0.01). In addition, compared to clinicians aged 20–39 years, both clinicians aged 40–59 years (*β* = 0.57, SE = 0.28, *z* = 2.04, *p* = 0.04) and clinicians aged 60–79 years rated significantly higher achievement (*β* = 0.75, SE = 0.39, *z* = 1.89, *p* = 0.059). The remaining main effects and interactions were not significant.

**Figure 2:**
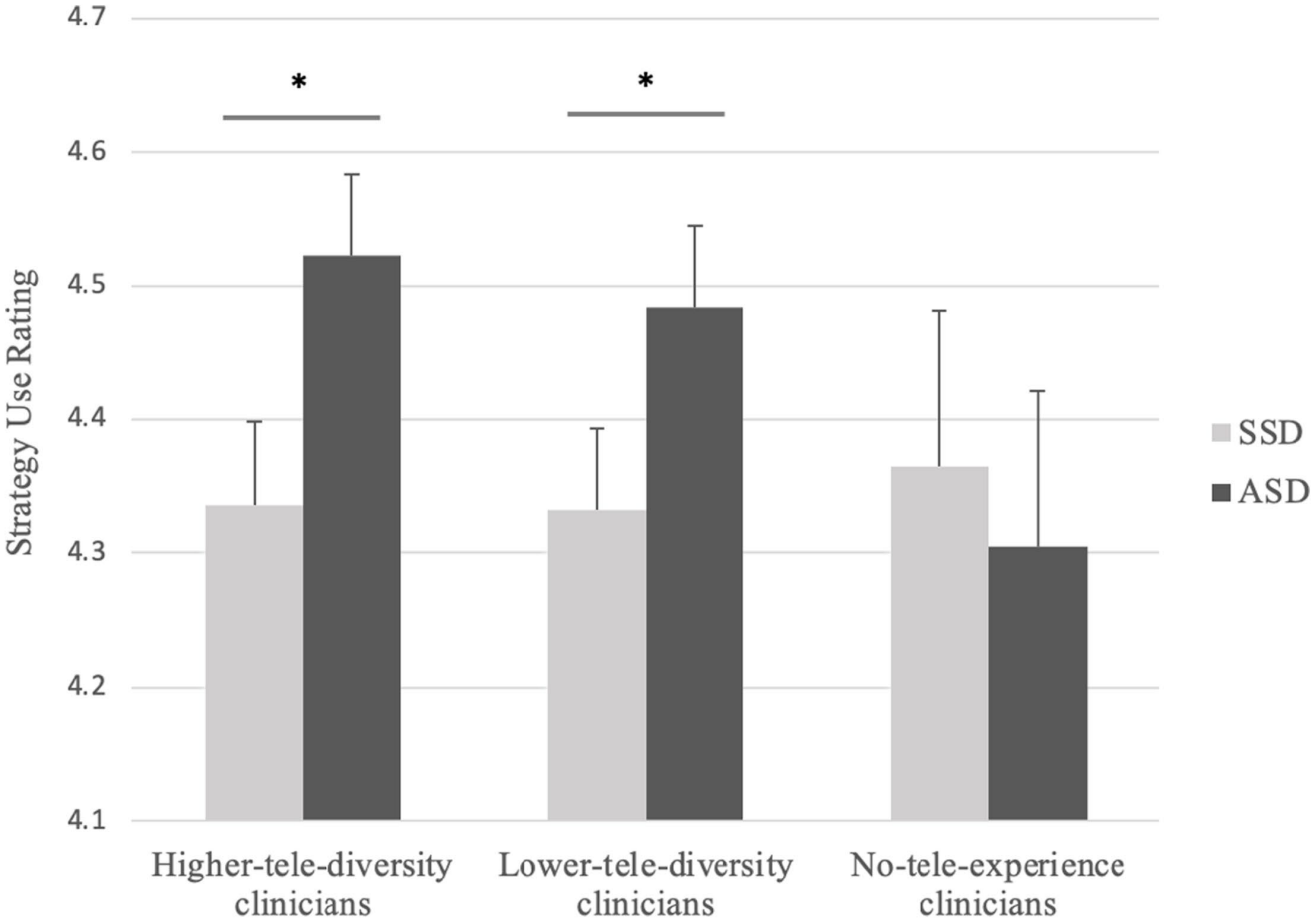
Interaction between clinician telehealth experience diversity and child diagnosis for strategy use in telehealth. ^*^indicates *p* < 0.01.

Regarding strategies of rapport building in telehealth, a model with by-participant intercepts and random slopes for child_disorder and child_age provided the best fit (AIC = 4652.0, *χ*^2^(3) = 45.54, *p* < 0.01). ASD received higher ratings than SSD (*β* = 0.73, SE = 0.23, *z* = 3.16, *p* < 0.01), and older children received lower ratings than younger ones (*β* = −1.32, SE = 0.22, *z* = −6.10, *p* < 0.01). In addition, clinicians aged 60–79 years rated higher in strategy use than 20–39 years (*β* = 0.69, SE = 0.29, *z* = 2.29, *p* = 0.02). Interestingly, there was a significant interaction between child disorder and clinician telehealth experience: the disorder effect (ASD > SSD) was present in the higher-telehealth-experience group (β = −0.75, SE = 0.16, z = −4.78, *p* < 0.01) and the lower-telehealth-experience group (β = −0.59, SE = 0.15, z = −4.02, *p* < 0.01), but was absent in the no-telehealth-experience group (β = 0.02, SE = 0.28, z = 0.06, *p* = 0.95). Figure 2 illustrates the interaction. All the other main effects and interactions were not significant.

### Correlations among aspects of rapport building

Each aspect score (importance, strategy use, achievement) was computed by averaging its constituent items, and each item score, in turn, was the mean of the four disorder-age conditions. It was found that clinicians’ ratings of rapport-building importance were positively and significantly correlated with reported strategy use (*r* = 0.384, *p* < 0.001), whereas strategy use did not significantly correlate with rapport-building achievement (*r* = −0.034, *p* = 0.626). Perceived importance was weakly but significantly associated with perceived achievement (*r* = 0.140, *p* = 0.044).

## Discussion

The study advanced the understanding of rapport building in telehealth speech-language services by specifying the influences of patient and therapist factors. Regarding patient factors, clinicians rated the ASD group higher importance, more strategy use, but lower achievement, compared to the SSD group. Clinicians reported more strategy use and lower achievement of rapport when serving younger children, but a main effect of child age was not evident in the importance of rapport. Regarding therapist factors, clinician age was related to the three aspects of rapport: older clinicians tended to rate rapport more important, reported higher achievement, and used more strategies than younger clinicians. Clinicians having telehealth experiences with the targeted children, regardless of higher or lower diversity, displayed ASD-SSD distinction in strategies employed, whereas such distinction was not found among those with no telehealth experience. Digital literacy was only related to perceived achievement of rapport building.

### Prevalent influences of child diagnosis and age on telehealth rapport building

As expected, clinicians rated children with ASD as requiring greater emphasis on rapport, using more strategies yet achieving lower levels of rapport than children with SSD. Younger children were reported to elicit more frequent strategy use, but a lower sense of achievement than older children, yet perceived importance was not significantly different between younger and older children. In general, these patterns are consistent with the diagnosis-specific and developmental profiles of pediatric clients. In particular, the diagnosis-related behaviors appeared to have more prevalent influences on clinicians’ rating of rapport. The increased cognitive and linguistic demands among children with ASD may complicate direct clinician-child interaction and increase reliance on caregivers as e-helpers. Further, digital literacy is likely required to overcome limitations in telecommunication, such as constrained visual field and signal latency in telehealth, reduced availability of subtle nonverbal cues, making it harder for clinicians to form a bond with the ASD group. Overall, these findings highlight not only the differing natures of ASD and SSD, but also the importance of developmental considerations when building rapport remotely.

Regarding the correlations among the three aspects of rapport building, clinicians who rated rapport as more important reported using more strategies, but greater strategy use may not translate into higher achievement in telehealth rapport. This is consistent with our prediction and underscores that rapport is a muti-faceted construct. The finding also suggests that the affective and behavioral facets of rapport may be selectively related rather than uniformly connected. This nuances Tickle-Degnen and Rosenthal (1990)‘s account, indicating that links between behavioral and affective facets may depend on which specific aspect of rapport is under consideration. Though not a main focus of the study, the weak but significant correlation between rapport-building importance and achievement likely reflects that both aspects tap the affective facet of the bond.

Future studies may go beyond the current focuses on client age and diagnosis to culturally and linguistically diverse populations with disabilities and explore how this factor influences telehealth rapport building. Currently, limited literature documented differences of perceptions of telehealth between monolingual and bi/multilingual children with communication disorders and their families. Davis et al. (2024) reported different perspectives between clients and clinicians: families of a bilingual background considered SLPs as a driving force during the therapy processes and heavily relied on SLPs for decision making, whereas SLPs considered their role as assistive. Though it is not specific to telehealth, this gap may cause a negative impact on rapport building. Nevertheless, telehealth offers unique opportunities to improve services for culturally and linguistically diverse populations who warrant greater attention (Taiebine and Keegan, 2024). They are frequently under- or over-referred in assessment, as monolingual clinicians often lack familiarity with home languages and cultural practices as well as with appropriate assessment tools and intervention materials (McLeod and Verdon, 2017; Scharff-Rethfeldt et al., 2020). Telehealth likely increases the access to bilingual clinicians and trained interpreters who are competent but not locally available. It also facilitates inclusion of family members as active e-helpers who can support interpretations of a child’s speech-language profile during assessment and intervention. In fact, positive evidence, using telehealth for bilingual children with ASD to complete a narrative language sample task (del Hoyo Soriano et al., 2021) and to provide AAC treatment for bilingual children (King et al., 2022), may support the acceptability of telehealth in this population.

The COVID-19 pandemic rapidly accelerated the use of telehealth in speech-language services (Learnihan et al., 2025). Though telehealth continued to be used, many services have since reverted to in-person care post-pandemic, so long-term effects of telehealth remain underexplored (Christopoulou et al., 2022). Insufficient clinician training in telehealth and limited experience in partnering effectively with parents continue to impede optimal delivery. Nonetheless, telehealth is well suited for disseminating brief, targeted training to clinicians, caregivers, educators, and policymakers, helping close research-practice gaps (Kim et al., 2024) and promote evidence-based, culturally responsive care (Petinou et al., 2024). For example, growing evidence indicates that bilingual exposure does not harm language development in children with ASD (Howard et al., 2024; Garrido et al., 2024), counters deficit-based recommendations to use only English at home (Martin Loya and Meadan, 2024; Pang, 2024), and may even confer some advantages associated with multilingualism (Gilhuber et al., 2023). Telehealth platforms are therefore well suited not only for service delivery but also for brief and targeted training to different stakeholders, thereby promoting more consistent care. Though our results provide limited insight into cultural and contextual influences on telehealth rapport building, future research should examine potential benefits and barriers across diverse cultural and linguistic contexts to inform more equitable and effective telehealth implementation.

### Different influences of therapist age, telehealth experience, and digital literacy

Compared to younger SLPs, older ones tended to rate higher in the importance, frequency of strategy use, and achievement of rapport in telehealth. The result appeared to contradict our prediction based on Tucker (2012a), which showed that older clinicians held more negative views of telehealth. However, in Tucker’s study, most SLPs had not yet started telehealth, whereas a majority of the clinicians in the present study had already adopted telehealth for the targeted children. We speculate that prior to the start of telehealth, older clinicians hold more negative views, which may prompt them to place greater emphasis and put more efforts into rapport building when they begin offering telehealth. In addition, the hands-on experience with telehealth could attenuate negative attitudes and increase their sense of achievement in rapport building. Future research may study the dynamic process by comparing clinicians’ perceptions of rapport before and after the initiation of telehealth.

Whether or not clinicians had telehealth experience significantly correlated with the frequency of their rapport building strategy use. Clinicians without telehealth experience with the targeted children reported similar levels of strategy use for both SSD and ASD, indicating a more uniform approach without emphasizing the influence of child diagnosis. In contrast, clinicians who had engaged in telehealth consistently reported higher strategy use for the ASD group compared to the SSD group. Clinicians with experience in telehealth should have experienced more difficulties with children with ASD than children with SSD, forcing them to develop more strategies that could be efficient in coping with the characteristics of ASD. By contrast, clinicians without hands-on telehealth experience lacked opportunities to test and consolidate rapport building strategies, so they may not differentiate their expected strategy use for ASD versus SSD. Also, the findings suggest that telehealth experience could be a key factor in developing strategies to build a strong rapport with children with ASD in telehealth, but not much with children with SSD. Interestingly, the ASD-SSD distinction pattern held irrespective of how many telehealth “cells” (i.e., 0–3-year-old SSD, 4–8-year-old SSD, 0–3-year-old ASD, or 4–8-year-old ASD) a clinician had worked with. Even those with fewer telehealth experiences matched the strategy use patterns reported by clinicians with more diverse telehealth backgrounds, indicating that even limited experience may yield insights into the need for enhanced or reduced frequency of strategies.

Clinician digital literacy was significantly correlated with their feelings of achievement, indicating that the competence with the internet and digital devices may translate into greater achievement of rapport in virtual sessions. Specifically, those who rated themselves higher in digital literacy possibly are better able to navigate telehealth platforms and troubleshoot technical issues, which enable them to devote more attention to client engagement. The client-centered interaction may help them feel more successful in fostering rapport and patient progress. By contrast, digital literacy did not have significant main effects on the perceived importance or strategy use of rapport in telehealth. One plausible explanation is that valuing rapport and selecting strategies may stem more from clinicians’ therapeutic beliefs, training, and clinical experience than from their comfort with digital interfaces.

### Recommendations to support digital literacy and telehealth rapport building

Given that only about 38% of respondents reported formal telehealth training, clinicians, especially those without hands-on telehealth experiences, are recommended to improve digital literacy and telehealth rapport-building skills through training that pairs technical skills (e.g., computer setup, platform features) with rapport-building techniques (e.g., brief caregiver coaching scripts, use of e-helpers). Unless having prior telehealth experience, clinicians should not provide telehealth guidance to caregivers. Clinicians may participate in supervised mock-sessions and peer observations by watching experienced telehealth clinicians and receiving constructive feedback, before independently implementing telehealth. In our study, verbal and non-verbal cues were used between frequently and always in both disorders and ages, and rating for non-verbal cues was slightly higher than verbal cues across all the disorder-age groups (Appendix 2). Grillo (2017) mentioned adaptations for non-verbal cues in telehealth, but it was unclear that the non-verbal cues were increased or decreased during telehealth. Presently, the results support the former, suggesting more frequent and exaggerated use of non-verbal cues to be detectable in telehealth that allows limited view.

Children with greater sensory and behavioral needs and of younger ages in particular require more intensive rapport-building strategies in telehealth and may nonetheless show lower immediate engagement. Caregivers therefore play a key facilitative role to help build rapport with therapists who work remotely. They are encouraged to prepare the child’s environment before sessions (e.g., quiet space, consistent seating, simple visual schedule), act as an active e-helper during sessions (e.g., follow the clinician’s cues, prompt turn-taking). Clinicians are encouraged to partner with caregivers by providing short, user-friendly guides (e.g., pictorial checklists, brief demo videos) that show exactly how to set up the camera and offer simple engagement prompts caregivers can use during the session.

For policymakers, community organizations, and patient-advocacy groups, system-level supports are needed to ensure access to telehealth services, especially in low-resourced areas. Policymakers and payers may consider reimbursement models and incentives that fund telehealth training and allow reimbursable preparatory time (e.g., caregiver coaching prior to a therapy session). Supported training should go beyond general platform use and basic telehealth knowledge to include focused, practice-based modules on clinically relevant, “trivial” aspects of remote care (e.g., rapport building techniques), so that clinicians can gain the specific skills needed to deliver high-quality telehealth services. Community organizations and advocacy groups can facilitate access by providing training to translate practical guides into local languages and low-literacy formats. It is important to prioritize early-intervention populations and children who have more severe behavioral and sensory challenges for enhanced supports.

### Limitations and future directions

At the time the survey was administered, we could not identify a validated instrument that measured the same construct in pediatric telehealth for speech-language service. Therefore, direct assessment of concurrent validity was not possible. The survey was derived from the extant literature, the best available evidence, and our clinical experience, and then refined through expert review. The instrument demonstrated good internal consistency. Confirmatory factor analysis of the prespecified three-aspect structure (importance, strategy use, achievement) offered modest support and suggests that further item refinement is needed to better distinguish among the three aspects. We acknowledge that the instrument requires additional psychometric validation, and that this is a limitation of the study that warrants continued efforts.

Digital literacy was operationalized narrowly, using two items that assessed clinicians’ competence of internet connection and device operation. These items provided limited information without capturing the broader and multi-dimensional competencies related to digital literacy. Therefore, future studies are warranted to employ validated and multi-item instruments to more comprehensively measure clinicians’ digital literacy and its relationship to telehealth. In addition, the low response rate (1.4%) may relate to selection bias which could limit the generalizability of the current findings. Clinicians who chose to participate in the survey may differ from non-responders. For example, the responders may have greater interests in telehealth, stronger buy-in to telehealth-based services, or higher digital literacy, which may not represent how the broader population perceives telehealth rapport building.

This study sampled SLPs practicing in the U.S., whereas many of the core lessons about remote rapport building (e.g., the importance of clinician digital literacy, the facilitative role of caregivers, and the need for rapport-focused clinical skills) are likely to resonate in other high-resource settings that have established telehealth infrastructure. Compared to major cities where resources are more abundant, telehealth has been less likely to be adopted in rural areas (Learnihan et al., 2025). In lower-resource settings, the same principles may still apply but require adapted delivery models, for example, low-bandwidth or phone-first workflows, community telehealth hubs or device-loan schemes, stronger emphasis on caregiver-mediated approaches, and medical insurance reimbursement covering telehealth. Overall, there is a lack of studies exploring telehealth in low-resource areas (Nizeyimana et al., 2022), warranting continued efforts in the generalizability of these findings in future research, particularly across different healthcare systems, resource settings, and cultural and linguistic contexts.

## Conclusion

The current study focused on how patient and therapist factors shaped clinicians’ perceptions of telehealth rapport building in pediatric speech-language services. The prevalent influences of child diagnosis and age highlight behavioral and developmental considerations pertaining to the client when clinicians need to build rapport remotely. Clinician factors influenced the three aspects of telehealth rapport differently. While clinician age was related to perceived importance, strategy use, and achievement of rapport, telehealth experience was only associated with the frequency of strategy use, and digital literacy was linked specifically to perceived levels of achievement. The three aspects capture different facets of the bond, and the affective and behavioral facets may not be uniformly connected. Together, the findings underscore the need to contextualize telehealth rapport by considering child and clinician factors, ultimately implying future training and practice in remote speech-language services and relevant disciplines where telehealth is frequently used to serving patients with disabilities.

## Data Availability

The raw data supporting the conclusions of this article will be made available by the authors, without undue reservation.

## Footnotes

1 There is no single consensus definition of “digital literacy” in the literature. For example, Mainz et al. (2024) treated it as an umbrella construct that includes technical competence (e.g., basic computer and software use, internet skills), methodological competence (e.g., data-handling and lifelong learning), social competence (e.g., digital engagement within teams and communities), and personal competence (e.g., reflective awareness of one’s digital skills). By contrast, Longhini et al. (2022) used the term more narrowly to denote competence in using technology (tablets, mobile phones, apps, internet), which corresponds closely to the technical competence component in Mainz et al. (2024). In this study, we followed the narrower definition, using “digital literacy**”** to refer primarily to practical skills related to internet connection and device operation.

2 Power analysis for GLMM was explored extensively, but there was a lack of guidance to address it (Johnson et al., 2015). It is worth noting that GLMM leads to greater power than ANOVA. Each participant responded to 32 questions regarding their perceived importance, strategy use, and achievement of rapport building, yielding 5,920 observations. All these observations were entered into the GLMM, whereas only averages of responses to each aspect of rapport building would be used in ANOVA.

3 The two questions captured different aspects of digital literacy (connecting to online services and operating devices such as computers, tablets, and smartphones), which has been brought up most frequently in the literature. In data analysis, as clinicians’ ratings of the two questions were strongly correlated, we averaged the scores and formed a single composite measure of overall digital literacy.

4 Note that each clinician provided rating of telehealth rapport building on 0–3 years old SSD, 4–8 years old SSD, 0–3 years old ASD, and 4–8 years old ASD, respectively.

5 The higher-diversity clinicians would have responded to the questions mostly based on their telehealth experience, whereas the lower-diversity clinicians may have partially predicted how they would establish rapport in telehealth based on limited experience with only one or two of these groups of children. The no telehealth experience group would have completely used predictions while responding to the questions.
