# Supplementary material for: Digital bonds: patient and therapist factors influence telehealth rapport building in speech-language services
Source: Front Psychol. 2025 Nov 27;16:1612803. doi: 10.3389/fpsyg.2025.1612803 (PMC12696860; doi:10.3389/fpsyg.2025.1612803)
Supplement: Supplementary file 1 [file Table_1.docx]

Appendix 1. Survey Questions

***Background Questions***

1. What is your gender?
   - Male
   - Female
   - Other (please specify): __________
2. What is your age?
   - 20 - 29
   - 30 - 39
   - 40 - 49
   - 50 - 59
   - 60 - 69
   - 70 - 79
   - Other (please specify): __________
3. Choose one or more races/ethnicities that you consider yourself to be (multiple choices allowed).
   - - Non-Hispanic White
     - Black or African American
     - Hispanic
     - Asian
     - Other (please specify): __________
4. What is the highest degree that you have obtained?
   - Master’s degree
   - PhD degree
   - Other (please specify): __________
5. What is your work setting (multiple choices allowed)?
   - - School
     - Early Intervention
     - College/University
     - Hospital
     - Residential Health Care (e.g., skilled nursing facilities)
     - Nonresidential Health Care (e.g., home health, outpatient)
     - Private Practice
     - Other (please specify): __________
6. What geographic region of the United States do you practice in?

- Northeast
- Southeast
- Southwest
- Midwest
- West

1. Would you define the geographic area in which you work as rural or urban?

- Rural
- Urban

1. How many years have you been a certified as a CCC-SLP?
   - 0 - 10 years
   - 11 - 20 years
   - 21 - 30 years
   - 31 - 40 years
   - More than 40 years
2. How many years have you used telepractice as a form of therapy intervention **in a pediatric setting**?

- Less than 1 year
- 1 - 2 years
- 2 - 3 years
- More than 3 years

1. What is your training regarding telepractice?
   - Formal training (e.g. took a course in college or a course with CE credit in telepractice)
   - No formal training (e.g., self-taught)
   - No training
2. On a scale of 1-5, rate your **overall competence of internet fluency** (not only specific to telepractice).
   - - 1 – Not at all competent
     - 2 – Low competence
     - 3 – Neutral
     - 4 – Competent
     - 5 – Very competent
3. On a scale of 1-5, rate your **overall competence** in regards to **using a computer, tablet, smart phone, etc**. (not only specific to telepractice).
   - - 1 – Not at all competent
     - 2 – Low competence
     - 3 – Neutral
     - 4 – Competent
     - 5 – Very competent

Caseload questions regarding different ages and populations differentiating entire working history caseload and telepractice only caseload. (Note: If you cannot remember how many children you have provided services to within the age ranges and disorders provided, please use your best estimate.)

1. How many children with this disorder and age have been on your **caseload for your entire working history**? (Try Your Best to Provide an Estimate)

Speech Sound Disorders

- - 0 Children
  - 1-10
  - 11-20
  - 21-30
  - 31-40
  - 41-50
  - 51-60
  - 61-70
  - 71-80
  - 81-90
  - 91-100
  - 100+

Autism Spectrum Disorder

- - 0 Children
  - 1-10
  - 11-20
  - 21-30
  - 31-40
  - 41-50
  - 51-60
  - 61-70
  - 71-80
  - 81-90
  - 91-100
  - 100+

1. How many children with this disorder and age have been on your **telepractice** caseload **only**?

Speech Sound Disorders

- - 0 Children
  - 1-10
  - 11-20
  - 21-30
  - 31-40
  - 41-50
  - 51-60
  - 61-70
  - 71-80
  - 81-90
  - 91-100
  - 100+

Autism Spectrum Disorder

- - 0 Children
  - 1-10
  - 11-20
  - 21-30
  - 31-40
  - 41-50
  - 51-60
  - 61-70
  - 71-80
  - 81-90
  - 91-100
  - 100

***Questions for Rapport Building in Telepractice***

Instructions: Please rate each of the statements below regarding **rapport building via telepractice** with children who have a diagnosis of speech sound disorders or children who have a diagnosis of autism spectrum disorders. For **each** of these two diagnoses, provide a rating for ages 0-3 and ages 4-8. Please remember that each of these diagnoses are primary diagnoses and not secondary to any other diagnosis.

**Importance of Rapport Building Within Telepractice Sessions**

Note: if you have not provided services to this age or population, please use your best estimate.

| ***Speech Sound Disorders***  (*Primary Disorder, Not Secondary)* | | ***Autism Spectrum Disorder***  (*Primary Disorder, Not Secondary)* | |  |
| --- | --- | --- | --- | --- |
|  | Ages 0-3 | Ages 4-8 | Ages 0-3 | Ages 4-8 |
| 15. Please rate the importance of rapport building for the success of telepractice sessions.  1 – Not at all important  2 – Low importance  3 – Neutral  4 – Important  5 – Very important | 1     2     3    4     5 | 1     2     3    4     5 | 1     2     3    4     5 | 1     2     3    4     5 |
| 16. Please rate how often you make rapport building a priority during telepractice sessions.  1 – Never  2 – Seldom  3 – Sometimes  4 – Frequently  5 – Always | 1     2     3    4     5 | 1     2     3    4     5 | 1     2     3    4     5 | 1     2     3    4     5 |
| 17. Please rate the necessity of training regarding telepractice rapport building prior to providing telepractice.  1 – Not at all necessary  2 – Low necessity  3 – Neutral  4 – Necessary  5 –Very necessary | 1     2     3    4     5 | 1     2     3    4     5 | 1     2     3    4     5 | 1     2     3    4     5 |

Note. Q17 was deleted in data analysis due to its low loading in confirmatory factor analysis, but still maintained here to present the complete survey questions that were presented to participants.

**Rapport Building Strategies Used Within Telepractice Sessions**

Note: if you have not provided services to this age or population, please use your best estimate.

|  | ***Speech Sound Disorders***  (*Primary Disorder, Not Secondary)* | | ***Autism Spectrum Disorder***  (*Primary Disorder, Not Secondary)* | |
| --- | --- | --- | --- | --- |
|  | Ages 0-3 | Ages 4-8 | Ages 0-3 | Ages 4-8 |
| 18. Please rate how often you need help from an **e-helper** (e.g., parent, grandparent, SLP assistant) to build rapport with clients via telepractice.  1 – Never  2 – Seldom  3 – Sometimes  4 – Frequently  5 – Always | 1     2     3    4     5 | 1     2     3    4     5 | 1     2     3    4     5 | 1     2     3    4     5 |
| 19. Please rate how often you use **verbal cues** (e.g., tone of voice, stressing important concepts, repeating information, listing information in sequential steps, etc.) to establish rapport in telepractice sessions.  1 – Never  2 – Seldom  3 – Sometimes  4 – Frequently  5 – Always | 1     2     3    4     5 | 1     2     3    4     5 | 1     2     3    4     5 | 1     2     3    4     5 |
| 20. Please rate how often you use **non-verbal cues** (e.g., eye contact, smiling, nodding, posture, etc.) to establish rapport in telepractice sessions.  1 – Never  2 – Seldom  3 – Sometimes  4 – Frequently  5 – Always | 1     2     3    4     5 | 1     2     3    4     5 | 1     2     3    4     5 | 1     2     3    4     5 |

**Rapport Building Achievement in Telepractice**

Note: if you have not provided services to this age or population, please use your best estimate.

|  | ***Speech Sound Disorders***  (*Primary Disorder, Not Secondary)* | | ***Autism Spectrum Disorder***  (*Primary Disorder, Not Secondary)* | |
| --- | --- | --- | --- | --- |
|  | Ages 0-3 | Ages 4-8 | Ages 0-3 | Ages 4-8 |
| 21. Please rate the **quality** of your rapport building via telepractice sessions.  1 – Very poor  2 – Poor  3 – Satisfactory  4 – High  5 – Very high | 1     2     3    4     5 | 1     2     3    4     5 | 1     2     3    4     5 | 1     2     3    4     5 |
| 22. Please rate the **speed** of your rapport building via telepractice sessions  1 – Very slow  2 – Slow  3 – Neutral  4 – Fast  5 – Very fast | 1     2     3    4     5 | 1     2     3    4     5 | 1     2     3    4     5 | 1     2     3    4     5 |

Appendix 2.

Clinicians’ perceived importance, strategy use, and achievement of rapport building in telehealth

| Aspects of Rapport Building | Disorder | SSD | | ASD | |
| --- | --- | --- | --- | --- | --- |
|  | Age | 0-3 y.o. | 4-8 y.o. | 0-3 y.o. | 4-8 y.o. |
|  | Measure | Mean (SD) | Mean (SD) | Mean (SD) | Mean (SD) |
| Importance | Rate the importance of rapport building for the success of telepractice sessions. | 4.72 (0.60) | 4.73 (0.49) | 4.70 (0.68) | 4.78 (0.57) |
|  | Rate how often you make rapport building a priority during telepractice sessions. | 4.34 (0.94) | 4.32 (0.86) | 4.39 (0.95) | 4.46 (0.85) |
|  | Rate the necessity of training regarding telepractice rapport building prior to providing telepractice. | 3.61 (0.98) | 3.50 (0.99) | 3.84 (1.00) | 3.85 (0.99) |
|  | Average | 4.23 (0.59) | 4.19 (0.54) | 4.31 (0.62) | 4.36 (0.55) |
| Strategy Use | Rate how often you use verbal cues to establish rapport in telepractice sessions. | 4.45 (0.79) | 4.44 (0.71) | 4.45 (0.87) | 4.46 (0.77) |
|  | Rate how often you use non-verbal cues to establish rapport in telepractice sessions. | 4.65 (0.65) | 4.62 (0.62) | 4.64 (0.69) | 4.66 (0.63) |
|  | Rate how often you need help from an e-helper to build rapport with clients via telepractice. | 4.42 (0.86) | 3.28 (0.91) | 4.66 (0.73) | 3.98 (0.87) |
|  | Average | 4.51 (0.57) | 4.11 (0.54) | 4.59 (0.58) | 4.37 (0.54) |
| Achievement | Rate the quality of your rapport building via telepractice sessions. | 3.51 (0.79) | 3.89 (0.78) | 3.29 (0.93) | 3.52 (0.89) |
|  | Rate the speed of your rapport building via telepractice sessions. | 2.83 (0.88) | 3.18 (0.83) | 2.52 (0.94) | 2.77 (0.87) |
|  | Average | 3.17 (0.72) | 3.54 (0.69) | 2.91 (0.81) | 3.15 (0.76) |

*Note*. The scales ranged from 1 to 5. See Appendix 1 for the complete questions and the descriptions of the 5 scales.
